# Intranasal Immunotherapy with M2 Macrophage Secretome Ameliorates Language Impairments and Autistic-like Behavior in Children

**DOI:** 10.3390/jcm13113079

**Published:** 2024-05-24

**Authors:** Ekaterina Ya. Shevela, Tatiana A. Loginova, Alexandr S. Munkuev, Tatiana E. Volskaya, Svetlana A. Sergeeva, Ivan M. Rashchupkin, Marina Yu. Kafanova, Valentina G. Degtyareva, Anastasia V. Sosnovskaya, Alexandr A. Ostanin, Elena R. Chernykh

**Affiliations:** 1 Laboratory of Cellular Immunotherapy, Research Institute of Fundamental and Clinical Immunology, 630099 Novosibirsk, Russia; iwwwanbets@mail.ru (I.M.R.); ostanin62@mail.ru (A.A.O.); ct_lab@mail.ru (E.R.C.); 2 Medical Center “Almadeya”, 194223 Saint-Petersburgh, Russia; lstatiana@mail.ru (T.A.L.); asmunkuev@gmail.com (A.S.M.); nushas@inbox.ru (T.E.V.); svetaslogoped@mail.ru (S.A.S.); 3 Department of Pediatrics, Medical Faculty, Federal State Budgetary Educational Institution of Higher Education “Novosibirsk State Medical University” of the Ministry of Health of Russia, 630091 Novosibirsk, Russia; kafanova@inbox.ru; 4 Medical Center “Sineglazka”, 630112 Novosibirsk, Russia; vd@sineglazka.com (V.G.D.); reg@sineglazka.com (A.V.S.)

**Keywords:** M2 type macrophages, cytokines, intranasal inhalations, specific language impairment, autism spectrum disorder, attention deficit hyperactivity disorder

## Abstract

**Background/Objectives**: The intranasal delivery of various neurotropic substances is considered a new attractive therapeutic approach for treating neuropathologies associated with neuroinflammation and altered regeneration. Specific language impairment (SLI) that arises as a result of damage to the cortical speech zones during the developmental period is one of the most common problems in preschool children, and it is characterized by persistent difficulties in the acquisition, understanding, and use of language. This study’s objective is to evaluate the efficacy and safety of intranasal immunotherapy using the M2 macrophage secretome as a rich source of immunoregulatory and neurotrophic factors for the treatment of severe language impairment in children. **Methods**: Seventy-one children (54 boys and 17 girls, aged 3 to 13 years) were recruited to participate in a clinical trial (NCT04689282) in two medical centers. The children were examined before, 1 month after, and 6 months after the start of therapy. In the vast majority of children (55/71), language impairment was associated with autistic-like symptoms and attention deficit hyperactivity disorder (ADHD). **Results**: Daily intranasal inhalations of M2 macrophage-conditioned medium (for 30 days) were well tolerated and led to a decrease in the severity of language impairments, autistic-like behavior, and ADHD symptoms. The clinical effect appeared within a month after the first procedure and persisted or intensified during a 6-month follow-up. Two-thirds of the children showed a clear clinical improvement, while the rest had less pronounced improvement. **Conclusions**: Thus, the use of the M2 macrophage secretome and its intranasal delivery is safe, well tolerated, and clinically effective in children with severe language impairments.

## 1. Introduction

Specific language impairment (SLI), also known as developmental language disorder, is one of the most common problems in the psycho-physical development of preschool children. SLI is characterized by persistent difficulties in the acquisition, understanding, production, and use of language that arise during the developmental period, typically during early childhood, as a result of damage to the cortical speech zones. This condition, which occurs in 5–10% of children, negatively affects the overall development of children’s personalities, their intellectual development, and their behavior, and causes significant limitations in an individual’s ability to communicate [1]. According to ICD-10, there is expressive language disorder (F80.1)—a disorder of the development of expressive speech, in which the child’s ability to use spoken language is noticeably below the level of the age norm with preserved speech understanding; and receptive language disorder (F80.2)—a disorder of the development of receptive speech, in which there is a misunderstanding of speech with intact hearing. Although SLI in children has been the focus of unceasing scientific attention for decades, the nature and mechanisms of this disorder remain unclear. In most cases, language disorders are associated with hypoxic-ischemic damage to the central nervous system (CNS) in the perinatal period [2].

Research over the past two decades has shown that the initiation of a neuroinflammatory process plays a central role in the pathogenesis of CNS damage [3]. Depending on the brain microenvironment, neuroinflammation may promote neurogenesis, which is typically observed in sudden injuries (traumatic brain injury, stroke), or negatively affect it, especially in chronic or excessive damage, impeding brain repair [4]. Therefore, certain prospects in the treatment of various CNS damage are associated with the use of cellular technologies, in particular those based on the use of progenitor cells and macrophages. These cells produce a large number of cytokines and growth factors that regulate the severity of the inflammatory response, on the one hand, and are involved in the “launch of” reparative processes, on the other hand [5,6,7]. However, the presence of the blood-brain barrier significantly limits the entry of cells and cytokines into the brain parenchyma when administered systemically. In this regard, the intranasal route of drug delivery, including cytokines, to brain tissue has attracted great interest in recent years.

The intranasal route of administration allows for the rapid penetration of various substances into the brain [8]. Experimental studies have shown that intranasally administered neurotrophic factors and erythropoietin quickly penetrate into brain tissue through the olfactory and trigeminal pathways, bypassing the blood-brain barrier [9,10,11]. At the same time, the demonstration of the clinical effects of intranasally delivered cytokines in models of various neuropathologies opens up broad prospects for introducing this approach into clinical practice [12,13].

Previously, we developed a new method for obtaining macrophages of the M2 phenotype, which are characterized by low antigen-presenting and pro-inflammatory activity while possessing a pronounced regeneration potential due to the high production of a variety of growth/trophic factors [14]. Indeed, the M2-conditioned media (M2-CM) contain a complex of cytokines, including neurotrophic, proangiogenic, and immunoregulatory factors. A clinical trial conducted in a group of 20 patients with organic brain lesions of various origins (NCT02957123, ClinicalTrials.gov) demonstrated good tolerability and safety of intranasal inhalations of M2-CM. Moreover, patients showed a statistically significant decrease in the level of anxiety and depression, as well as corrections of cognitive impairment. The clinical effect was maintained during 6 months of observation. Based on the above, the aim of this study was to assess the safety and clinical effectiveness of the intranasal administration of the conditioned media of M2 macrophages in children with severe language impairments associated with perinatal or postnatal lesions of the central nervous system.

## 2. Materials and Methods

### 2.1. Study Design

Our clinical trial was carried out according to the IFCI-20/06/2017 protocol, approved by the decision of the local ethics committee of the Research Institute of Basic and Clinical Immunology, and registered on the website www.ClinicalTrails.gov (NCT04689282). The study recruited 71 children (54 boys and 17 girls), aged 3 to 13 years (Me 6.0, IQR 4.7–7.0). The children have perinatal-related language disorders (72%, 51/71) or postnatal (28%, 20/71) lesions of the central nervous system of various origins (hypoxia, trauma, infection, intoxication, post-vaccination complications, etc.), verified by a speech therapist and neurologist, with written informed consent from the parents. The study did not include children with acute infectious diseases, convulsions, gentamicin intolerance, and/or multiple drug allergies. Children whose language impairment was explained by hearing loss, mental retardation, severe somatic pathology, malnutrition, as well as the influence of unfavorable social factors (insufficient communication and education) were also excluded from the recruited group.

### 2.2. Outcomes

The children’s development was assessed by a neurologist, speech therapist, and parents before the therapy (visit 1), one (visit 2) and six (visit 3) months after the start of therapy. The primary endpoint was the change in the severity of language disorders according to a speech therapy examination. For this, we used a modified point-level language development scale (LDS) [15]. The scale includes six series of tests aimed at research: I—speech understanding (maximum score 33), II—sensorimotor level of speech (maximum 38 points), III—grammatical structure of speech (maximum 24 points), IV—vocabulary and word formation skills (maximum 17 points), V—coherent speech (maximum 25 points), VI—general and fine motor skills (maximum 23 points). Based on the maximum number of points (160), the test execution percentage (PET) was calculated as the total score for the test/160 × 100. Completing tests at 80–100% indicates level IV of language impairment; the child speaks quite well and his or her speech practically does not differ from peers. A score of 65.0–79.9% indicates level III, a mild delay. These children can make sentences, but their semantic and sound loads are not sufficiently developed. A score of 50.0–64.9% indicates level II, a moderate delay. These children present an initial development of general speech, but their vocabulary remains poor. A score of 49.9% or less indicates level I, a severe delay. These children completely lack coherent speech. PET scores were also calculated for each series (number of points awarded per series × 100/maximum possible score for a given series).

The secondary endpoints were: (1) the safety assessment, including analysis of adverse reactions (allergic, toxic, inflammatory reactions, neurological deterioration, convulsive syndrome) during the therapy with the soluble M2 factors; (2) the assessment of autism spectrum disorder (ASD) symptoms; (3) the assessment of attention deficit hyperactivity disorder (ADHD); (4) an assessment of the satisfaction of the patient’s parents with the treatment on a 5-point Likert scale from 1 (“very dissatisfied”) to 5 (“very satisfied”). When assessing their satisfaction with treatment, parents had to answer several questions: How effective do you think your child’s treatment is? How concerned are you about the side effects of treatment? How satisfied are you with the safety of the treatment? How willing are you to continue treatment if necessary? How satisfied are you with the overall duration of treatment? Would you recommend the same treatment to someone else with a similar condition? As a result, parents gave an integral assessment of their satisfaction with the treatment on a 5-point Likert scale as follows: very satisfied; satisfied; neutral; not satisfied; very unsatisfied.

To assess the ASD symptoms, the CARS rating scale for autism and autism-like conditions in children was used. Children with total scores in the range of 37–60 or 30–36 were diagnosed as having severe or mild/moderate ASD, respectively.

The SNAP-IV scale was used to assess attention deficit hyperactivity disorder (ADHD). The ADHD diagnosis criteria were >25 points for boys and >22 points for girls. ADHD severity was determined separately for boys and girls. (For boys: 26–34 points—mild; 35–43 points—moderate; 44–54 points—severe degree. For girls: 23–31 points—mild; 32–40 points—moderate; 41–54 points—severe degree.)

Additionally, we asked parents to provide a subjective assessment of the severity of their children’s language disorders and symptoms of ASD. For this, we used questionnaires in which parents had to note the most striking positive symptoms that appeared after treatment, which we took into account.

### 2.3. Conditioned Media of M2 Macrophages

Conditioned media of M2 phenotype macrophages (M2-CM) were prepared in accordance with [16]. For this, M2 macrophages were generated from mononuclear cells (MNCs) obtained from a parent’s peripheral blood by density gradient centrifugation (Ficoll-Paque, Sigma-Aldrich, St. Louis, MO, USA). The MNCs were incubated in tissue culture flasks (TPP, Trasadingen, Switzerland) for 18 h, followed by the removal of non-adherent cells. The adherent fraction of MNCs (containing at least 93–95% of CD14+ monocytes) was then cultured in RPMI-1640 medium supplemented with 0.05 mM 2-mercaptoethanol, 2 mM sodium pyruvate, 0.3 mg/mL L-glutamine, 1% non-essential amino acids (all reagents from Sigma-Aldrich, Burlington, NJ, USA), 100 μg/mL gentamycin, 2% autologous plasma, and recombinant human GM-CSF (rhGM-CSF, 50 ng/mL, Sigma-Aldrich, Burlington, NJ, USA). On day 7, the conditioned media of the macrophage cultures was collected, subjected to sterilizing filtration, aliquoted into sterile vials (2.0 mL/vial), and stored at −20 °C until use. The median protein concentration in the M2 macrophage supernatants was 1.09 g/L (from 1.0 to 1.27 g/L).

### 2.4. Inhaled Immunotherapy (IIT)

Before use, the M2-CM (2.0 mL) was thawed at room temperature and used intranasally with a compressor inhaler, once a day, for a course of 30 days. The first 2–3 inhalations were carried out under the supervision of a physician, and the physician monitored the patient’s systemic (temperature, pulse, heart rate, respiratory rate, partial pressure of oxygen, skin rash, cough, etc.) and local (nasal itching, epistaxis, decreased or altered sense of smell and taste, sinusitis, etc.) reactions for two to three days. Subsequent treatment was carried out on an outpatient basis, and parents had to notify the physician of any adverse reactions. Blood parameters (leukocytosis, ESR, CRP, fibrinogen, and other indicators) were not evaluated because, in children, even a simple blood draw presents certain difficulties and, in most cases, is poorly tolerated by the child. IIT was carried out after completing the next course of basic medical and nonmedical therapy, which included nootropic and vascular drugs, vitamins, dehydration and anticonvulsant therapy, massage, laser therapy, etc. For daily intranasal utilization, 2 mL of M2 product containing 2 mg of protein was used.

### 2.5. Statistical Analysis

Statistical analysis was performed using the Statistica 6.0 software package. The data were presented as median (Me) values and interquartile ranges (IQR, 25–75% quartiles). To assess the significance of differences, the nonparametric Mann–Whitney U-test was used. Kruskal–Wallis one-way analysis was used to compare median values across multiple groups. We considered a result of *p* < 0.05 to be statistically significant. The evaluation of the significance of the initial total score on the language development scale in predicting an early (1 month) response to IIT was carried out using ROC analysis (Receiver Operating Characteristic analysis) using the GraphPad Prism 5.0 program.

## 3. Results

### 3.1. Characteristics of Children Included in the Study

The children were examined and treated at two medical centers, in Novosibirsk (Sineglazka Medical Center, n = 32) and St. Petersburg (Almadeya Medical Center, n = 39). In all of the children, language impairment manifested itself as a specific language impairment (ICD-10 code: F80). The majority of children (57/71, 80%) demonstrated the absence of coherent speech (level I language impairment); 9 participants (12.5%) had initial manifestations of coherent speech, characterized by poor vocabulary and agrammatism phenomena (level II); and only 5 children (7.5%) demonstrated a mild delay in language development (level III).

In the vast majority of children (55/71, 77%), language impairment was associated with autistic-like symptoms, while half of these children additionally exhibited symptoms of ADHD (26/55). Evaluation on the CARS scale demonstrated the signs of moderate (Me 31; IQR 30–34 points) and severe (Me 42; IQR 38–45 points) ASD in 30 (54%) and 25 (46%) children, respectively. In total, 34 children were diagnosed with ADHD, with a mean SNAP-IV score of 34.5 (IQR 31–39), among them 14 had mild ADHD and 20 had moderate/severe ADHD.

All of the children were characterized by behavioral problems (motor stereotypies, emotional lability with affective reactions and inadequacy of the degree of expression of the emotional response, insufficient formation or lack of self-care skills, including the inability to dress or eat independently) and attention impairments (quick satiety, increased distractibility, impaired auditory attention).

An assessment of language development before the therapy revealed pronounced impairments in all components of speech (Table 1). The lowest test execution percentage was recorded for series III, IV, and V (on average from 0 to 4.4%). PET for “speech understanding” (series I) and “sensorimotor level of speech” (series II) averaged 15.2% and 19.7%, respectively. Finally, the children successfully completed only 47.8% of the tests of “general and fine motor skills” (series VI). The median total score was 38.0 out of a maximum possible 160 (PET—Me 23.9%). In the majority of children (57/71, 80%), the test execution percentage did not exceed 49.9% (Me 18.0%, IQR 7.5–28%), which indicated a severe delay in language development (level I, non-speaking children). In the remaining 20% of cases, language impairments were moderate (10/71) or mild (4/71) (level II and III). Notably, the boys, who made up the majority of the study cohort, did not differ from girls in the manifestations of ASD (33 (28–39) vs. 35 (29–38) on the CARS scale, *p* = 0.49) and ADHD (34 (27–38) vs. 33 (24–35) on the SNAP-IV scale, *p* = 0.45) but the boys were characterized by less pronounced language impairments, while girls were characterized by significantly more severe language delays (43 (20–72) vs. 19 (7–45) on the LDS, *p* = 0.04).

### 3.2. Early Effects of Inhaled Immunotherapy

All children recruited into the study received a 30-day course of inhaled immunotherapy (IIT) in the form of daily intranasal inhalations of conditioned M2 media (M2-CM). IIT was well tolerated and did not cause any unwanted side effects (including allergic, toxic, and inflammatory reactions) or neurological deterioration. In two cases, parents noted short-term nasal congestion and the appearance of mucous discharge, which resolved independently and did not require medications. In two other cases, intranasal administration was accompanied by sneezing. In one child, the parents noticed sleep worsening when inhalations were performed in the evening. However, with inhalations in the morning, the child’s sleep returned to normal.

On the second visit, i.e., one month after the start of the IIT, no statistically significant changes in speech activity were detected in the whole group of examined children (n = 71) (Table 2). However, the children showed a decrease in the severity of autistic-like behavior, as evidenced by a decrease in the CARS score from 33.0 to 29.0 (*p* = 0.002). In addition, children with signs of ADHD showed a decrease in the total score on the SNAP-IV scale from 34.5 to 28.5 (*p* = 0.011).

Despite the absence of significant changes in the LDS in the whole cohort of children, an analysis of individual values revealed the heterogeneity of the clinical response to the therapy. Thus, according to the results of speech therapy testing, in 66% of cases (in 47/71 children, subgroup “Responders”), a significant improvement in speech activity was recorded, which was manifested by an increase in total PET of more than 5% (Me 16.5%; IQR 10.5–32.0%). In the remaining 34% of cases (in 24/71 children, subgroup “Non-responders”), one month after the start of therapy, the increase of the total LDS score was less than 5% (Me 0%; IQR 0–4.0%), indicating the absence of any dynamics of language impairment. The children in these groups were similar in age, with a median age of 6 years in each group. Notably, the number of responders among boys was significantly higher (40/54, 74%) than among girls (7/17, 41%, p_FET_ = 0.0187), which can be explained by the less severe language impairments in the boys at baseline. Among the children who responded to IIT, a particular group exhibited a very evident clinical response (a 30% or greater increase in total PET). Notably, these children were younger than the other group, aged 4.9 years compared to 6.3 in the former group (*p* = 0.009). 

Evaluation of parents’ satisfaction with the treatment on a 5-point Likert scale showed that even at such an early follow-up stage (1 month after the start of the IIT), the vast majority were either very satisfied (39/71, 55%) or generally satisfied (24/71, 34%) with the result obtained. In only 11% of cases (8/71) did parents show a neutral attitude toward the therapy.

In addition, all parents filled out two questionnaires, in which they noted the most striking positive changes in speech and autism-like symptoms. The data in Table 3 shows that during the treatment, parents noted an increase in their children’s speech activity in approximately 50% of cases, in 38–42% an improvement in active and/or passive vocabulary, in 42% of cases the child more often initiated communication, and also could speak a lot of words and construct phrases. Approximately every fifth treated child had improved expressiveness and/or intelligibility of speech, and the number of words in a sentence increased.

Parents also recorded obvious positive dynamics in autistic-like symptoms (Table 3). Approximately half of the treated children, one month after the start of therapy, showed improved contact with people, appropriate expression of feelings, the ability to repeat words and/or actions, and an improvement in general intelligence. In a third of children, parents noted improved speech, more adequate use of toys and/or gestures, and a decrease in fear and nervousness. Parents noticed the children became calmer, their levels and stability of attention increased, self-care skills began to develop or improved (the ability to eat, dress independently, etc.), and phrasal speech appeared.

### 3.3. Long-Term Effects of Inhaled Immunotherapy

In a separate group of 39 children, who were examined 1 and 6 months after the start of therapy, the stability of the clinical effects of IIT was analyzed. From the data in Table 4, it is clear that in this group no statistically significant changes in speech activity were detected 1 month after the start of the IIT. However, a significant decrease in the severity of autistic-like behavior was recorded, as evidenced by a decrease of the CARS score from 35.0 to 28.0 (P_U_ = 0.03). Compared to the initial level, the results of speech therapy testing after 6 months showed an average of 23.5% improvement in speech understanding; a 73% improvement in the sensorimotor level of speech, a more than twofold improvement of word formation skills; and a 40% improvement of general and fine motor skills (P_U_ = 0.03). The most difficult to correct was the ability to form coherent speech (series V). As a result, the overall LDS score significantly increased from the initial 45.5 to 64.5 points, and the test execution percentage improved from 28.4% to 40.3% (P_U_ = 0.03). Testing on the CARS and SNAP-IV scales did not reveal any changes compared to previous (at 1 month) results, indicating the stability of positive changes in autistic-like behavior and ADHD symptoms.

Analysis of the individual values 6 months after the start of IIT showed that, compared with the initial level, almost half of the children (19/39, 49%) showed a marked improvement in speech activity, which was manifested by an >30% increase of the total PET score (Me 58, 0; IQR 42.0–100.0). In 46% of cases (18/39 children) a moderate clinical response was recorded as evidenced by a <30% increase of the PET score (Me 17.5; IQR 13.0–23.0). Notably, in only 5% of cases (2/39) were no dynamics of speech activity noted.

When parents were surveyed at the third visit (6 months after the start of IIT), the vast majority were either very satisfied (23/39, 59%) or generally satisfied (13/39, 33%) with the therapy outcome. In only 8% of cases (3/39) did parents show a neutral attitude toward the therapy.

The results of a survey of parents regarding the changes in language impairments and autism-like symptoms are presented in Table 5. It can be seen that 6 months after the start of treatment, parents noted an increase in the child’s speech activity, plus an improvement in active and/or passive vocabulary in 82% of cases. In 66% of cases, the child more often initiated communication, and in 59%, the intelligibility of words and the ability to use phrasal speech increased.

Some obvious positive dynamics were also recorded in autistic-like symptoms (Table 5). Actually, parents noticed their children’s improved contact with people, more adequate expression of emotions, reduction of increased nervousness, improvement in general intelligence (in 59% of cases), the ability to repeat words and/or actions (in 90% of cases), improved speech (in 74% of cases), etc.

None of the parents expressed regret about their participation in this clinical study. Moreover, most of them expressed a desire to take a second IIT course.

Thus, the positive effect of intranasal immunotherapy on language impairments, autistic-like behavior, and ADHD symptoms was found to persist and even intensify six months after the start of the course of treatment. A comparison of the median values using a one-way Kruskal–Wallis analysis of variance showed highly significant differences in the speech activity (% PET, H = 9.166, *p* = 0.010), CARS score (H = 16.209, *p* = 0.0003) and SNAP-IV score (H = 10.148, *p* = 0.006) during the six month follow-up period.

### 3.4. Predictors of Early Clinical Response to IIT

We have shown that intranasal inhalations with M2 macrophage secretome allow 66% of children (47/71, “Responders”) to achieve a significant clinical effect beginning just one month after the start of therapy (see Section 3.2). However, in 34% of cases (24/71, “Non-responders”), children did not respond to immunotherapy. Considering these data, it seemed important to compare the initial (before the start of the IIT) parameters of the children who did show a clinical response versus those who did not. Such a retrospective analysis could serve as the basis for identifying the most informative diagnostic signs (predictors) that could make it possible to predict the effectiveness of IIT.

The children in both groups did not differ significantly in age, average CARS, and SNAP-IV scores. However, the results of speech therapy testing showed (Table 6) that children who did not respond to IIT initially differed from the opposite group in that they had more severe language impairments, which was manifested by a 3-fold difference in the LDS total score (Me 18.5 vs. 54.0, *p* = 0.0001).

**Figure 1:**
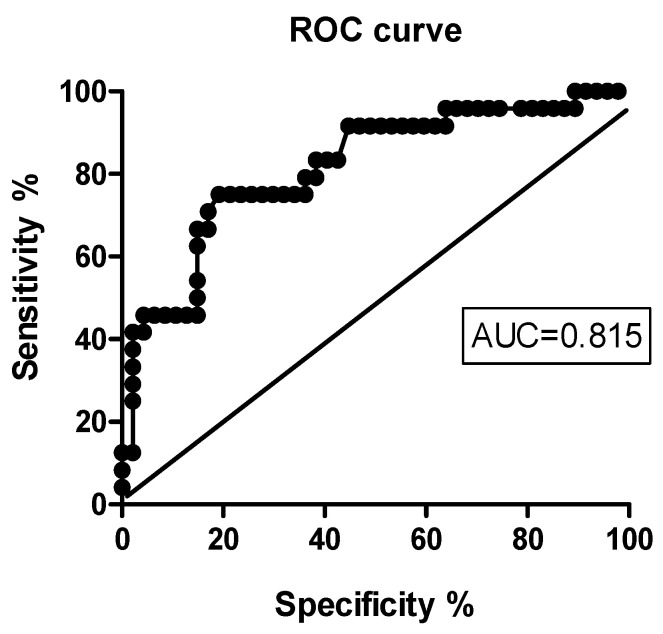
ROC curve illustrating the relationship between sensitivity and specificity for various cutoff points of the total LDS score in predicting clinical response to IIT.

These results became the basis for analyzing the operational characteristics of the prediction of an early (at 1 month) clinical response to IIT, based on determining the total score on the LDS before the therapy. For this, a characteristic curve (receiver-operator curve, ROC) was constructed. Figure 1 shows that the area under the curve in this case was 0.815 (95% CI 0.70–0.92, *p* = 0.0001).

The ROC curve was constructed to determine sensitivity, specificity, and likelihood ratios for various cutoff points. For the total LDS score, the cutoff point corresponding to the maximum sensitivity (81.0%) and specificity (75.0%) was a threshold value of >24.6 (likelihood ratio 3.23), which made it possible to highly predict the achievement of a significant clinical outcome effect by the end of the course of therapy (1 month after the start of IIT). With initial LDS scores < 22.5, an early clinical response to IIT seems unlikely (SP 83.0%; SN 71.0%; Likelihood ratio 4.16). However, even in this case, the improvement in the child’s speech activity may be delayed and appear later (after 6 months).

## 4. Discussion

This study was, to our knowledge, the first to examine the effect of chronic intranasal immunotherapy with M2 macrophage secretome in children with language impairments associated with perinatal or postnatal lesions of the central nervous system. A clinical study has shown that intranasal inhalations of soluble M2 macrophage factors are safe, well tolerated, and clinically effective in the treatment of children with severe language impairments. Intranasal immunotherapy leads to (1) a decrease in the severity of language impairments (which is manifested in a 45% improvement in speech understanding, a 51% improvement in the sensorimotor level of speech, and a 72% improvement in word formation skills); (2) a twofold increase in general and fine motor skills, and (3) a decrease in autistic-like symptoms and manifestations of ADHD. The clinical effect appears quite quickly—within a month after the first procedure—and persists or intensifies during a 6-month follow-up. It is noteworthy that two-thirds of the children showed a clear clinical improvement, while the rest had less pronounced improvement. In addition, we determined the threshold value of the initial total score on the speech development scale (24.6 points), which allows us to predict the achievement of an early significant clinical effect with a high probability (sensitivity 81% and specificity 75%).

Currently, the treatment of CNS diseases using cell-based approaches is rapidly expanding, especially approaches based on the use of stem cells [17]. At the same time, macrophages, namely macrophages of the M2 phenotype, represent another type of cell that plays a key role in the healing of various tissues, including the repair of nervous tissue, by reducing the detrimental effects of inflammatory factors and creating a microenvironment that promotes neuronal regeneration [18]. The first experimental evidence indicating the possible involvement of macrophages in neuroregeneration was published in 1993, when Hikawa N. et al. demonstrated in vitro that uptake of cellular debris induces macrophages to produce factors that stimulate neuronal survival and axonal regeneration [19]. Currently, macrophages are highly attractive for cell-based therapy compared to stem cells since they are non-oncogenic, non-teratogenic, highly secretory (therefore, much of their regenerative potential has been attributed to paracrine functions), and, finally, macrophage-based therapy may be simply repeated and is not related to any ethical problems.

To investigate the therapeutic potential of human macrophages with the M2 phenotype, we designed an original protocol for obtaining M2-like macrophages from circulating monocytes based on their interaction with apoptotic cells [20]. Generated macrophages expressed M2-associated and pro-apoptogenic molecules; produced low levels of pro-inflammatory cytokines and a high concentration of TGF-b; were unable to stimulate effective T-lymphocyte proliferation and Th1/Th17 responses; produced various growth, neurotrophic, and angiogenic factors with higher levels of IGF-1, VEGF, and EPO as compared to M1 macrophages; and demonstrated neuroprotective and neuroregenerative activity in vitro and in vivo [20,21,22]. We evaluated these macrophages and their soluble factors in several pilot clinical trials. In particular, we demonstrated the good tolerability and safety of intranasal inhalation of M2 macrophage soluble factors in patients with organic brain lesions (NCT02957123, ClinicalTrails.gov).

With the strong belief that the therapeutic effect of macrophage-based therapy relies primarily on the macrophage secretome, we analyzed macrophage-conditioned media for 54 analytes, and managed to demonstrate that a M2 macrophage culture medium offers a rich source of a wide range of neurotrophic, neuroprotective, and angiogenic factors (ΕPO, VEGF, IGF-1, BDNF, EGF, FGF-basic et al.) [16,20,23]. It is noteworthy that the range of growth and trophic factors produced by M2 phenotype macrophages is in many ways similar to that of mesenchymal stromal cells as well as neural stem cells [24]. VEGF and IGF-1 were found to be most actively produced at high levels by M2 macrophages. Along with its key role in vasculo-/angiogenesis, VEGF is directly or indirectly involved in the regulation of neurogenesis, stimulating the proliferation of neuronal precursors [25,26]. The neuroregenerative properties of VEGF have been demonstrated both in vitro and in various CNS lesions in vivo [27,28]. When delivered intracerebroventricularly, VEGF stimulated neurogenesis and angiogenesis, which led to a decrease in the infarct area [29]. The effects of VEGF may also be mediated by stimulating brain-derived neurotrophic factor (BDNF) production by endothelial cells [30]. Further, increasing literature has confirmed the anti-inflammatory properties of VEGF [31,32]. Another factor, IGF-1, plays a key role in vascular remodeling but also stimulates the growth and survival of neurons [33]. Additionally, IGF-1 promotes the maturation and function of oligodendrocytes [34]. Actually, administration of IGF-1 reduces the death of nerve cells in a model of ischemic brain injury [35].

It is very important to mention that most cytokines and growth factors identified in the M2 secretome are involved in the regulation of neural cell function and survival. In addition, due to their multiple interactions, the effects of the macrophage secretome, as a mixture of multiple soluble molecules, cannot be explained by the individual factors/cytokines. A similar conclusion was made by Wang, who demonstrated that some cytokines (TGF-α/β, PDGF, EGF, and IGF-1) in platelet-rich plasma (PRP) might benefit, while others might induce negative effects (VEGF, TNF-α, Ang-1, and SDF-1α) or no effects (CTGF, FGF, HGF, PF4, and KGF) in osteoathritis. Nevertheless, PRP definitely has a positive effect [36].

Notably, the therapeutic potential of the macrophage-conditioned medium may be mediated not only by soluble factors, but also by microvesicles and exosomes, which play an important role in cell-to-cell communication [37,38]. Zhan et al. demonstrated that macrophage-derived exosomes promote nerve regeneration both in vitro and in vivo [39]. In another study, administration of M2 macrophage exosomes significantly inhibited glial scar formation [40]. We did not evaluate exosomes and microvesicles in the M2 conditioned media used for intranasal delivery, which is one of the limitations of our study. However, given the therapeutic effect of macrophage-derived exosomes and their capacity to induce M2 polarization in microglia [41], we cannot exclude the involvement of extracellular vesicles in the clinical effects we observed.

Over the last few years, there has been a growing interest in the intranasal delivery of therapeutic substances for treating disorders affecting the CNS. The intranasal administration allows for rapid absorption and effective penetration of various substances into the brain [8] and has a number of advantages, including ease of administration, a rapid onset of action, the possibility of repeated administration, minimal delivery time, no need for modification of cytokines to overcome the blood-brain barrier, and the absence of systemic side effects [9,10]. Moreover, intranasal delivery overcomes several problems of oral administration, such as systemic toxicity, hepatic first-pass metabolism, and gastrointestinal enzymatic degradation [42]. Finally, intranasal delivery ensures patient comfort, which is extremely important for pediatric patients.

Experimental studies have shown that intranasally administered neurotrophic factors quickly penetrate into brain tissue through the olfactory and trigeminal pathways, bypassing the blood-brain barrier [9,10,11]. The penetration of substances (for example, insulin or IGF-1) from the nasal cavity into brain tissue has been reported in humans [43,44]. Neurological improvements with intranasally administered cytokines in various models of neuropathology open up broad prospects for the translation of this approach into clinical settings [45,46]. In fact, the intranasal route of delivery is being intensively developed for neurodegenerative diseases (Alzheimer’s disease, Parkinson’s disease, etc.), where the lack of effective treatment is an important current problem, and neurodevelopmental conditions (for example, autism spectrum disorders), which currently have no approved medication [47]. Indeed, intranasal administration of the neuropeptide oxytocin resulted in alleviating social shortfalls in children with autism spectrum disorders [48,49,50].

Many studies, preclinical and clinical, show the ability of intranasal insulin to improve cognition and memory in age-related cognitive deficits (summarized in [51]) and neurodevelopmental disorders [52], in particular in children suffering from 22q13 deletion syndrome, which is associated with cognitive impairments and autistic behavior [53].

Noteworthy, as of March 2024, 23 clinical trials including the terms “nasal” and “neurodevelopmental disorders in children”, were listed on ClinicalTrials.gov. Among them, 16 trials aim to explore the neural and/or behavioral effects of oxytocin in autism spectrum disorders, and only nine of them reported the results obtained. There are no clinical trials on cell-based or cell-free therapy for this group of pathologies. This situation can only be explained by the fact that, regardless of the great therapeutic importance of the intranasal delivery route for children with neurodevelopmental pathology, the field is in its infancy. Clinical investigations with a higher level of evidence are certainly necessary for the subsequent translation of the therapeutic approach we propose into clinical practice, particularly randomized placebo-controlled multicenter trials.

## 5. Conclusions

To summarize the above, we have for the first time tested a fundamentally new approach based on the use of the M2 macrophage secretome and the intranasal route of its administration for the treatment of children with severe language impairments. The data obtained indicate the safety and effectiveness of macrophage-based inhaled immunotherapy.

## Figures and Tables

**Table 1:** Language development of children at baseline (n = 71).

Language Development Scale, LDS, Series	Score Me (IQR)	The Test Execution Percentage, PET (%) Me (IQR)
Series I (speech understanding) (max 33 points)	5.0 (1.0–21.0)	15.2 (3.0–63.6)
Series II (sensorimotor level of speech) (max 38 points)	7.5 (2.2–23.7)	19.7 (5.9–62.5)
Series III (grammatical structure of speech) (max 24 points)	0 (0–5.0)	0 (0–20.8)
Series IV (vocabulary and word formation skills) (max 17 points)	0.7 (0–6.0)	4.4 (0–35.3)
Series V (coherent speech) (max 25 points)	0 (0.0–2.5)	0 (0–10.0)
Series VI (general and fine motor skills) (max 23 points)	11.0 (5.0–16.0)	47.8 (21.7–69.6)
Total score (max 160 points)	38.0 (16.0–65.0)	23.9 (10.0–40.8)

**Table 2:** Change in LDS, CARS, and SNAP-IV one month after the beginning of therapy.

Parameters Me (IQR)	Visit 1 (at Baseline, n = 71)	Visit 2 (in 1 Month, n = 71)	P_U_
LDS, series			
Series I (speech understanding) (max 33 points)	5.0 (1.0–21.0)	5.2 (1.5–22.0)	0.47
Series II (sensorimotor level of speech) (max 38 points)	7.5 (2.2–23.7)	11.7 (3.0–27.7)	0.26
Series III (grammatical structure of speech) (max 24 points)	0 (0–5.0)	0 (0–8.0)	0.55
Series IV (vocabulary and word formation skills) (max 17 points)	0.7 (0–6.0)	1.0 (0–7.0)	0.48
Series V (coherent speech) (max 25 points)	0 (0.0–2.5)	0 (0–2.5)	0.95
Series VI (general and fine motor skills) (max 23 points)	11.0 (5.0–16.0)	12.5 (6.0–18.0)	0.23
Total score (max 160 points)	38.0 (16.0–65.0)	42.7 (18.2–75.7)	0.29
PET, %	23.9 (10.0–40.8)	26.7 (11.4–47.3)	0.29
CARS, points	33.0 (28.0–39.0)	29.0 (24.0–33.0)	0.002
SNAP-IV, points	34.5 (31.0–39.0)	28.5 (24.0–32.0)	0.011

Note: LDS, Language development scale. PET, The test execution percentage. CARS, the CARS rating scale for autism and autism-like conditions. SNAP-IV, the SNAP-IV scale for attention deficit hyperactivity disorder. P_U_—significance of differences between visits 1 and 2 (U—nonparametric Mann–Whitney test).

**Table 3:** Results of a parent survey one month after the start of the IIT.

What Are the Most Striking Positive Symptoms, in Your Opinion, That Appeared after Treatment:	n	%
*Language impairments*		
increasing speech activity	38/71	53
2. the child is more often the initiator of communication	30/71	42
3. active vocabulary (what the child says) has improved	27/71	38
4. passive vocabulary has improved (what the child understands)	42/71	59
5. speech intelligibility has increased	13/71	18
6. speech expressiveness has improved	15/71	21
7. grammatically correct speech construction has improved	10/71	14
8. the number of words in a sentence has increased	17/71	24
9. the number of sentences for telling about something has increased (detailed answer)	8/71	11
10. approximately how many words can your child currently speak: speaks a lot of words; builds sentences/phrases	30/71	42
*Autism-like symptoms*		
11. improving contact with people	38/71	53
12. the ability to repeat words, actions, etc.	38/71	53
13. more adequate expression of emotions	38/71	53
14. decrease in severity/disappearance of “pathological” movements	17/71	24
15. more appropriate use of toys	21/71	29
16. improved reaction to change	34/71	48
17. improved ability to look at objects or make eye contact	30/71	42
18. faster and more appropriate response to sounds	30/71	42
19. the child become more adequate in such actions as touching, sniffing, tasting, and responding to pain	25/71	35
20. reduction of fear and nervousness	23/71	32
21. speech improvement	27/71	35
22. more adequate use of gestures and improvement of their understanding	25/71	35
23. normalization of the child’s activity (decrease in hyperactivity or increase in initially low activity)	15/71	21
24. improvement of general intelligence (corresponding to the intelligence of children of the appropriate age)	34/71	48

**Table 4:** Change in LDS, CARS, and SNAP-IV one and six months after the beginning of therapy.

Parameters Me (IQR)	Visit 1 (at Baseline)	Visit 2 (1 Month)	Visit 3 (6 Month)
LDS series			
Series I (speech understanding) (max 33 points)	17.0 (2.0–25.0)	18.0 (3.0–33.0)	21.0 (4.0–33.0)
Series II (sensorimotor level of speech) (max 38 points)	10.7 (2.7–22.2)	13.0 (5.7–27.5)	18.5 (7.0–29.5)
Series III (grammatical structure of speech) (max 24 points)	0 (0–5.0)	1.2 (0–8.0)	2.7 (0–10.0)
Series IV (vocabulary and word formation skills) (max 17 points)	2.2 (0–8.0)	3.5 (0–8.0)	5.0 (0–10.2)
Series V (coherent speech) (max 25 points)	0 (0–3.5)	0 (0–5.0)	0 (0–5.0)
Series VI (general and fine motor skills) (max 23 points)	10.0 (5.0–14.7)	12.5 (7.2–17.2)	14.0 * (9.0–18.5)
Total score (max 160 points)	45.5 (24.0–68.5)	58.7 (26.7–83.2)	64.5 * (33.2–89.0)
PET (%)	28.4 (15.0–42.8)	36.7 (16.7–52.0)	40.3 * (20.8–55.6)
CARS, points	35.0 (27.0–41.0)	28.0 * (23.0–34.0)	27.0 ** (21.0–33.0)
SNAP-IV, points	32.5 (27.0–38.0)	29.0 (22.0–35.0)	28.0 * (19.0–31.0)

Note: * *p* < 0.05; ** *p* < 0.01—significance of differences compared to baseline values (U—nonparametric Mann–Whitney test). n = 39. PET, the test execution percentage (%).

**Table 5:** Results of a parent survey six months after the start of the IIT.

What Are the Most Striking Positive Symptoms, in Your Opinion, That Appeared after Treatment:	n	%
*Language impairments*		
increasing speech activity	32/39	82
2. the child is more often the initiator of communication	26/39	66
3. active vocabulary (what the child says) has improved	32/39	82
4. passive vocabulary has improved (what the child understands)	32/39	82
5. speech intelligibility has increased	23/39	59
6. speech expressiveness has improved	10/39	25
7. grammatically correct speech construction has improved	10/39	25
8. the number of words in a sentence has increased	20/39	51
9. the number of sentences for telling about something has increased (detailed answer)	10/39	25
10. approximately how many words can your child currently speak: speaks a lot of words; builds sentences/phrases	23/39	59
*Autism-like symptoms*		
11. improving contact with people	23/39	59
12. the ability to repeat words, actions, etc.	35/39	90
13. more adequate expression of emotions	23/39	59
14. decrease in severity/disappearance of “pathological” movements	6/39	15
15. more appropriate use of toys	16/39	41
16. improved reaction to change	19/39	49
17. improved ability to look at objects or make eye contact	19/39	49
18. faster and more appropriate response to sounds	19/39	49
19. the child become more adequate in such actions as touching, sniffing, tasting, and responding to pain	13/39	33
20. reduction of fear and nervousness	23/39	59
21. speech improvement	29/39	74
22. more adequate use of gestures and improvement of their understanding	23/39	59
23. normalization of the child’s activity (decrease in hyperactivity or increase in initially low activity)	6/39	15
24. improvement of general intelligence (corresponding to the intelligence of children of the appropriate age)	23/39	59

**Table 6:** Initial (before treatment) parameters in children opposite in early (after 1 month) clinical response to IIT.

Parameters Me (IQR)	Responders (n = 47)	Non-Responders (n = 24)	P_U_
Age, years	6.0 (4.4–7.0)	6.0 (5.0–7.0)	0.71
CARS, points	33.0 (27.0–38.0)	35.0 (30.2–40.2)	0.14
SNAP-IV, points	36.0 (26.0–39.0)	34.0 (28.5–36.5)	0.80
Language development scale			
Series I (max 33 points)	16.0 (4.5–24.5)	1.0 (0.1–2.0)	0.0001
Series II (max 38 points)	16.0 (6.0–25.5)	2.9 (1.2–13.7)	0.004
Series III (max 24 points)	2.0 (0–10.2)	0 (0.0–0.6)	0.016
Series IV (max 17 points)	3.0 (0–8.0)	0 (0–0)	0.0001
Series V (max 25 points)	0 (0–5.0)	0 (0–0)	0.005
Series VI (max 23 points)	12.0 (6.0–16.0)	6.5 (3.0–13.6)	0.08
Total score (max 160 points)	54.0 (30.0–82.0)	18.5 (4.7–31.0)	0.0001

Note: P_U_—significance of differences between groups (U—nonparametric Mann–Whitney test).

## Data Availability

Data are available from the corresponding author upon reasonable request.
